# Association of Allostatic Load and All Cancer Risk in the SWAN Cohort

**DOI:** 10.3390/cancers14133044

**Published:** 2022-06-21

**Authors:** Jie Shen, Bernard F. Fuemmeler, Yufan Guan, Hua Zhao

**Affiliations:** 1 Departments of Family Medicine and Population Health, School of Medicine, Virginia Commonwealth University, Richmond, VA 23284, USA; jie.shen@vcuhealth.org (J.S.); yufan.guan@vcuhealth.org (Y.G.); 2 Departments of Health Behavior and Policy, School of Medicine, Virginia Commonwealth University, Richmond, VA 23284, USA; bernard.fuemmeler@vcuhealth.org

**Keywords:** allostatic load, cancer risk, chronic stress

## Abstract

**Simple Summary:**

Chronic stress has been linked to increased risks for many chronic diseases. However, its contribution to cancer risk is still unclear. In this study, we analyzed the association between allostatic load (AL), a biomarker of chronic stress, and cancer risk, in the Study of Women’s Health Across the Nation (SWAN). We found that women with the highest levels of AL had a 64% increased risk of overall cancer. And the association was independent of demographics, healthy behaviors, and socioeconomic factors. In summary, we provide evidence that chronic stress may increase the risk of cancer.

**Abstract:**

Elevated chronic stress is thought to increase cancer risk, though the results so far have been inconsistent. In this study, we assessed the relationship between allostatic load (AL), a biological indicator of chronic stress, and overall cancer risk in 3015 women who participated in the Study of Women’s Health Across the Nation (SWAN). Based on the distribution of AL, the study population was categorized into four groups, from the lowest (1st category) to the highest AL group (4th category). At baseline, African American and Hispanic women were more likely to be in the higher AL categories than White women (*p* < 0.001). In addition, women who smoked regularly, drank alcohol regularly, had no leisure physical activity, and had restless sleep were also more likely to be in the higher AL categories than their relative counterparts (*p* < 0.001). We also observed that women in the lower-income category with no health insurance were more likely to be in the higher AL category (*p* < 0.001). The study then found that women in the 4th category of AL (the highest AL group) had a 1.64-fold increased risk of overall cancer (Hazard ratio (HR): 1.64, 95% confidence interval (CI): 1.04, 2.59). The risk association was further strengthened after adjusting demographics, healthy behaviors, and socioeconomic factors with an HR of 2.08. In further analysis of individual biomarkers of AL score, we found that higher levels of triglyceride and CRP were associated with increased risk of cancer, highlighting the role of metabolic dysfunction and inflammation in the etiology of cancer development. In summary, we report that higher AL is associated with increased cancer risk.

## 1. Introduction

The greater ‘wear and tear’ on the body due to increased chronic stress has long been speculated as a cancer risk factor. Such an assumption is biologically plausible. Chronic stress can weaken the immune system [1], and a comprised immune system plays a role in cancer development [2]. Chronic stress can alter the levels of certain hormones [3], which may increase the risk of developing cancer [4]. Chronic stress can also lead to unhealthy behaviors [5]. Overeating, physical inactivity, smoking, and heavy drinking are all lifestyle factors that increase cancer risk [6]. However, the past epidemiological studies have been mixed and tend to be null [7,8,9,10,11,12,13,14]. These inconsistent results may be partly attributable to the difficulty of quantifying chronic stress objectively and biologically meaningfully. It may also be partly due to the weaknesses in epidemiological study design. Thus, to date, the relationship between chronic stress and cancer risk is still largely unclear.

Allostatic load (AL) score, a multi-system, multi-dimensional composite index that usually involves cardiovascular, metabolic, immunological, and neuroendocrine components, has been demonstrated to be an adequate assessment of the health impact of chronic stress levels [15]. Compared to many other stress measurements, AL describes the physiological burden of cumulative stress and accounts for individuals’ response and adaptation to the stress burden. Thus, it is less subjective and more biologically relevant. In addition, AL is socially patterned and a predictor of morbidity and mortality of many age-related diseases [16]. Several epidemiological studies explored the role of AL in cancer outcomes, ranging from tumor characteristics [17,18] to survival [19,20]. For example, we previously reported that a higher AL score was associated with increased odds of having poorly differentiated breast tumors [18]. A positive association was found between the AL score and cancer-specific mortality [19]. Furthermore, in a cross-sectional study using the data from NHANES 1999–2008, elevated AL was found associated with a history of breast cancer among Black women [21]. However, there is no prospective study to examine the association between AL score and cancer risk.

In this study, using longitudinal data from the Study of Women’s Health Across the Nation (SWAN) [22], we assessed the association between pre-diagnosed AL and cancer development during the follow-up. We hypothesized that higher AL was associated with increased cancer risk. In addition, we also evaluated the relationship between AL with demographics, healthy behaviors, and socioeconomic factors at baseline.

## 2. Methods and Materials

### 2.1. Study Population

We acquired the data for this study from the Study of Women’s Health Across the Nation (SWAN), a multi-center study of women’s health through menopausal transition comprising a baseline evaluation and ten waves of following annual evaluations [22]. The study eligibility included: (1) age (42–52 years old), (2) with an intact uterus and at least one ovary, (3) not using exogenous hormone preparations affecting ovarian function in the month before the baseline interview, as well as no hormone use in the three months before study screening; (4) with at least one menstrual period in the last three months. The study was approved by the institutional review board (IRB) at each SWAN study site. At baseline, a total of 3302 women met the eligibility criteria. All the factors essential for the AL score construction was available from 3015 women. Therefore, they were included in the final study sample. The cancer diagnosis was self-reported during the follow-up.

### 2.2. AL Score Construction

We used 11 factors to construct the AL score from measures collected at the baseline data collection period. The detailed methods of measurement of factors were described by Chyu et al. [23]. Those factors are well-recognized markers of cardiovascular (systolic (SBP) and diastolic blood pressure (DBP)), inflammatory (C-reactive protein (CRP)), metabolic (high and low density lipoprotein cholesterol (HDL and LDL), total cholesterol, waist to hip ratio, fasting serum glucose, and triglycerides), and neuroendocrine (dehydroepiandrosterone (DHAS)) systems. We included the history of taking medication to control metabolic diseases and hypertension to account for the medication. We combined LDL and total cholesterol to generate a new “abnormal cholesterol” factor. Cases with total cholesterol >240 mg/dL or total cholesterol ≥240 mg/dL and LDL >130 mg/dL were deemed to have abnormal cholesterol. Thus, our AL score included a total of ten factors. In this study, we used a cutoff value to assign each variable a threshold of risk that determined the score (0 or 1) that each variable would contribute to the computed AL score (Table 1) [18]. Then, points were totaled to obtain a continuous measure for AL, each with a maximum possible score of 10 (0–10). The score was then categorized into four groups based on the distribution of the score (Table 2).

## 3. Statistical Analysis

Statistical analyses were performed using the Stata software package (version 13, StataCorp, College Station, TX, USA). Descriptive statistics were applied to each demographic, lifestyle, and socioeconomic factor. Women who smoked regularly were defined as those who have ever smoked a total of at least 20 packs of cigarettes over a lifetime or at least one cigarette per day for at least one year. Women who drank alcohol regularly were defined as having at least one alcoholic drink per month. Women who had no leisure physical activity were defined as those who did not play any sports or exercise in the past year. ANOVA was applied to assess the difference across four categories of AL. Association between cancer risk and AL score was assessed using univariate and multivariable-adjusted Cox proportional hazards regression models. Adjusted hazard ratios (HRs) and 95% confidence intervals (95% CIs) were estimated, and potential confounding factors were adjusted as appropriate. To explore which components of AL were related to cancer risk we repeated these analyses, examining each biomarker of the AL within adjusted hazard regression model. All statistical tests were two-sided, and *p* values of less than 0.05 were considered statistically significant.

## 4. Results

Eleven factors were used, including SBP, DBP, HDL, LDL, total cholesterol, triglycerides, waist to hip ratio, blood glucose, CRP, DHAS, and a history of medication controlling metabolic diseases and hypertension. For individual biomarkers initially reported as continuous variables, predetermined cutoff points were applied to categorize the patients into high and low-risk groups (Table 1). Over 10% of cases had elevated SBP and DBP. About one-third had an abnormal HDL. Approximately 24% of patients had a waist to hip ratio of at least 0.85. About 11% had elevated blood glucose. Over a third had higher serum CRP levels. In terms of DHAS, 9.12% had an increased risk. In addition, about 4% of women took medication to control metabolic diseases and hypertension.

Overall, the median calculated AL score was 1 (Table 2), ranging from 0 to 9. On one side, no women had all 10 risk factors (AL = 10). On the other hand, approximately 30% of the women had no risk factor (AL = 0), and 25% had only one risk factor (AL = 1). Given the distribution of the AL score, we divided the study population into four categories. Category 1 (lowest) included women with an AL score of 0 (29.35%), Category 2 included women with an AL score of 1 (25.01%), Category 3 included women with an AL score of 2 to 3 (30.85%). Category 4 (highest) included women with an AL score of 4 to 9 (14.65%).

We investigated whether the distributions of demographics, healthy behaviors, and SES factors differed among AL score categories (Table 3). A statistically significant trend of increasing mean age from AL Category 1 to 4 was observed (*p* = 0.004). The distribution of race/ethnicity significantly differed among AL score categories (*p* < 0.001). With the increase of AL score category from 1 to 4, the percentage of African American and Hispanic women in each category increased, respectively. Meanwhile, the proportion of White, Chinese American, and Japanese American women in each category decreased, respectively. Regarding healthy behaviors, the distributions of cigarette smoking, alcohol consumption, leisure physical activity, and self-rated sleep quality differed significantly by AL score category (*p* < 0.001, respectively). Specifically, with the increase of AL score category from 1 to 4, the percentage of women who smoked regularly, drank alcohol regularly, had no leisure physical activity, and had a restless sleep in each category increased, respectively. On the other hand, the percentage of women who never smoked regularly, never drank alcohol regularly, had leisure physical activity, and had sound and restful sleep in each category decreased, respectively. Family income and health insurance status differed significantly by AL score category (*p* < 0.001, respectively). With the increase of AL score category from 1 to 4, the percentage of women with less than 20 k per year, having 20–50 k per year and having no health insurance in each category increased, respectively. Meanwhile, the percentage of women with at least 100 k per year, 50–100 k per year, and health insurance in each category increased, respectively.

**Figure 1:**
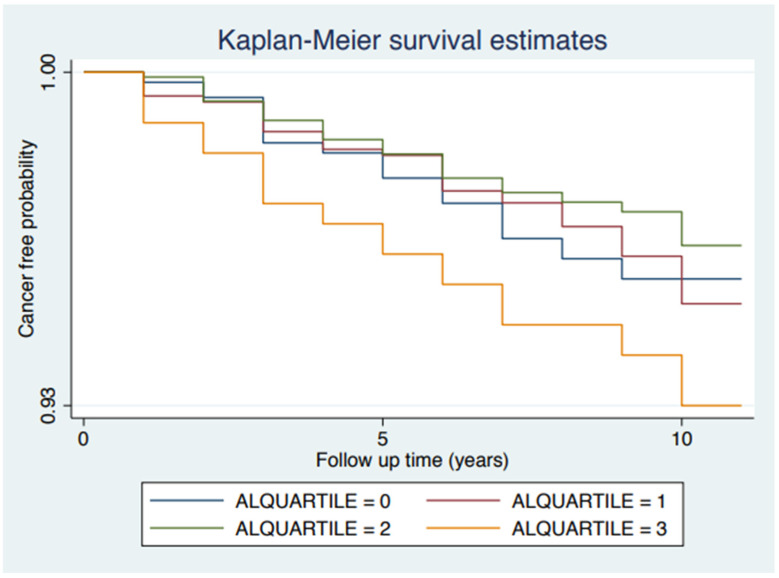
Shows the Kaplan–Meier survival estimates for the association between the AL score category and overall cancer risk (*p* < 0.001).

During the follow-up, 149 women developed cancer. The distribution of cancer cases differed by AL score category (*p* = 0.037). Compared to 4.62% in Category 1, 7.45% were observed in Category 4. We investigated the relationship between the AL score category and cancer risk (Table 4). Compared to category 1 (lowest AL scores), in the univariate Cox regression analysis, category 4 (highest AL scores) was significantly associated with increased risk of overall cancer (HR = 1.64, 95% CI: 1.04, 2.59). Neither category 2 nor 3 was associated with the risk of overall cancer. Figure 1 shows the Kaplan–Meier survival estimates for the association between the AL score category and overall cancer risk (*p* < 0.001). In further multivariate analysis, we included age and race/ethnicity in Model 1; age and race/ethnicity, smoking, alcohol consumption, leisure physical activity, and sleeping quality in Model 2; and age and race/ethnicity, smoking, alcohol consumption, leisure physical activity, and sleeping quality family income and health insurance in Model 3. The association between AL score Category 4 and overall cancer risk remained statistically significant in all four models (Model 1: HR = 1.88, 95% CI: 1.17, 3.02; Model 2: HR = 2.09, 95% CI: 1.29, 3.41; and Model 3: HR = 2.08, 95% CI: 1.26, 3.42). In a further trend test, with the increase of AL category from 1 to 4, a statistically significant increasing trend was observed for models 3 and 4 (*p* for trend 0.031 and 0.040, respectively). To further confirm the association, we treated the AL score as a continuous variable. Increased AL score was associated with a 1.13-fold increased risk of cancer (HR = 1.13, 95% CI: 1.03, 1.25).

Finally, we explored the association between individual biomarkers of AL score and cancer risk (Table 5). After the adjustment of demographics, healthy behaviors, and SES factors, we found that higher levels of triglycerides and CRP were associated with 1.68 and 1.42 folds increased risk of overall cancer (triglycerides: HR = 1.68, 95% CI: 1.16, 2.43; CRP: HR = 1.42, 95% CI: 1.01, 2.01).

## 5. Discussion

Previous studies showed that increased levels of AL are associated with aggressive tumor characteristics [17,18] and shorter survival [19,20] among cancer patients. However, prior to this report, the association between AL and cancer risk has not been assessed. In this study, we reported that women with the highest AL scores had an increased risk of cancer. We also found that AL was significantly affected by demographics (e.g., age and race), healthy behaviors (e.g., cigarette smoking, alcohol consumption, leisure physical activity, and sleep quality), and socioeconomic factors (e.g., family income and health insurance). In addition, we reported that among all biomarkers of AL score, increased levels of triglyceride and CRP were associated with increased risk of cancer.

Our findings that higher AL is associated with increased cancer risk are not surprising. Prior reports suggested that increased AL disrupt the nervous system and the stress response axis [24,25], resulting in the disturbance of immune, cardiovascular, metabolic, and neuroendocrine systems, and further promoting tumorigenesis [26]. In addition, an elevated AL, as an indicator of higher levels of chronic stress, may also indirectly promote carcinogenesis by inducing excessive stress hormones (e.g., catecholamines and glucocorticoids) [4] and increased DNA damage and genomic stability [27], which are also hallmarks of cancer. Furthermore, in our previous study in breast cancer patients, we found a significant positive correlation between AL with leukocyte mitochondrial DNA copy number variation (*p* < 0.001) [18]. The role of mitochondrial in cancer development has been well-documented [28] and thus, together with the current study, it is possible that the observed association between AL and future cancer risk may be mediated by such processes.

Intriguingly, our study found that among all biomarkers of AL score, higher levels of triglycerides and CRP were associated with 1.68 and 1.42 folds increased risk of cancer (triglycerides: HR = 1.68, 95% CI: 1.16, 2.43; CRP: HR = 1.42, 95% CI: 1.01, 2.01). The relationship between circulating glyceride and cancer risk has been reported previously [29,30]. In the metabolic syndrome and cancer project, Borena et al., reported that the relative risk of top quintile versus bottom quintile of triglycerides of overall cancer was 1.16 (95% CI: 1.06–1.26) in men and 1.15 (95% CI: 1.05–1.27) in women [29]. The role of CRP in cancer etiology is well-documented [31]. In our previous study in Mexican Americans, we reported that study participants in the 4th quartile with the highest CRP levels had a significantly 1.88-fold increased risk of cancer (HR = 1.88, 95% CI: 1.12, 3.13) compared to those in the 1st quartile with the lowest CRP levels [32]. In addition, marginally significant association was observed for HDL and waist to hip ratio (HDL: HR = 1.33, 95% CI: 0.96, 1.85; CRP: HR = 1.38, 95% CI: 0.95, 1.99). Among the four components of the AL score, our results provide evidence to support the notion that metabolic and immunological components are the potential biological pathways linking AL and cancer development. However, due to the small sample size, we may not see the significant associations between other components of AL with cancer risk if the strength of the associations is modest. Thus, the contribution of other components of AL to cancer risk cannot be ruled out.

Chyu et al., assessed the relationship between demographics and socioeconomic factors with AL score in a sample of non-Hispanic White, African American, Chinese, and Japanese women identified from the SWAN [23]. Though we used different cutoff points to define the risk group for each AL score biomarker and included the history of medication to control metabolic diseases and hypertension, the results are generally in agreement. For example, significant racial differences in AL score between African American and White women and a significant trend of decreasing AL score with the increase of family income were observed in both studies. However, unlike Chyu’s study, our study included Hispanic women. Compared to their White counterparts, we found that Hispanic women had significantly higher AL scores (2.38 vs. 1.51, *p* < 0.001) and were more likely to be in the higher AL score category. A previous study by Peek found that Hispanics had a higher AL score than Whites, but the difference was not statistically significant [33]. They further reported that the AL score differed by born place among Hispanics. American-born Hispanics had a higher AL score than foreign country-born Hispanics. Unfortunately, nativity information for Hispanic women was not assessed in this study.

Unhealthy behaviors, including smoking, excessive drinking, disturbed sleep, and physical inactivity, can promote and aggravate pathophysiology by dysregulating key biological components involved in AL. Thus, as expected, in our study, we found that women who ever smoked regularly, had no leisure physical activity, and had a restless sleep were more likely to be in the higher AL categories than their relative counterparts (*p* < 0.001). Several studies support the link between smoking and high AL [34,35,36,37,38]. In addition, a few studies identified that higher physical activity is associated with decreased AL [39,40,41,42,43]. Thus, our results are consistent with the literature reports. Using a subset of SWAN study participants (*N* = 330), Hall et al., assessed the relationship between chronic stress (measured using upsetting life events) with subjective and objective sleep outcomes [44]. They reported that chronic stress is prospectively associated with sleep disturbance. Though we used different measures for chronic stress, our results are consistent with their findings.

A few studies showed that moderate alcohol consumption has beneficial effects in lowering AL in men and women [45,46,47,48]. In our study, we confirmed the association. Women who drank alcohol moderately were found to be more likely to be in the lower AL category compared to their counterparts (*p* < 0.001). In our study, most women who drank alcohol moderately reported as having fewer than two drinks per week (80.89%), indicating that most of them who reported drinking alcohol were low-to-moderate alcohol users. Low-to-moderate alcohol use has been known to lower the risk of metabolic syndrome compared with abstainers. In addition, blood pressure is either positively or neutrally affected by low-to-moderate amounts of alcohol [49]. Given metabolic and blood pressure measures are critical components of AL, it is not surprising that alcohol use appeared to confer lower AL in our sample.

Another interesting finding in this study is that the significant association between AL score and cancer risk was not diminished but enhanced by adjusting demographics, healthy behaviors, and SES factors. Intriguingly, many of those factors influenced the AL score. The demographics (e.g., age and race) and healthy behaviors (e.g., cigarette smoking, alcohol consumption, leisure physical activity, and sleep quality) included in this study are well-known cancer risk factors. Though SES factors (e.g., family income and health insurance) may not directly promote cancer development, lower-income and no health insurance may prevent women from accessing health care, cancer screening, preventive measures, adopting a healthy lifestyle, and ultimately increasing cancer risk. Thus, our results which showed the significant association between AL and cancer risk was not affected by those factors, suggest that at least partially, AL may reflect the biological pathways linking those common cancer risk factors and cancer development. In addition, the findings also indicate that AL (with components assessing HDL and CRP) may be a useful biomarker predictive of cancer risk.

There are some limitations to this study. Due to the age limits (42 to 53 years old) of the SWAN study participants and relative short follow-up time (10 years), the number of cancer cases is small (Supplementary Materials). Thus, we did not have the statistical power to perform stratified analysis to assess the association for specific cancers. There is no consensus of how to how to construct the AL score [17,21,50,51,52,53,54,55]. Therefore, we cannot rule out that different results may be produced if we choose a different way to construct the AL score. However, there is a general agreement that immune, cardiovascular, metabolic, and neuroendocrine systems must be represented in any AL score. This is the case in our study. Additionally, previous studies showed that despite variances in the construction of AL score, results are generally in agreement [56,57]. Given the age limitation of the SWAN study population, the results obtained from this study may not be replicated in women in other age groups. It would be interesting to confirm the findings from this study in other extensive studies with women from all age groups. Nevertheless, the considerable strengths of our study outweigh the limitations. 

## 6. Conclusions

In summary, we carried out the first study to evaluate the association between AL and cancer risk in a multi-ethnic women cohort. Findings from this study contribute essential knowledge to the role of chronic stress and its biomarker, AL, in the etiology of cancer development. We believe AL presents an opportunity to be used as a biomarker for stress reduction-based cancer prevention. For example, AL can be used as a biomarker to monitor the effect of mindfulness-based stress reduction trials among high-risk individuals and cancer survivors. Additional research with large sample sizes is needed to further validate these novel findings.

## Figures and Tables

**Table 1:** Distribution and high-risk cutoff points for individual biomarkers of AL scores *.

Biomarkers	Mean (SD)	Cutoff Value	N (%) at Risk
SBP (mm Hg)	118.23 (17.24)	≥140	377 (11.45)
DBP (mm Hg)	75.57 (10.80)	≥90	394 (11.98)
HDL (mg/dL)	55.90 (14.56)	<50	1173 (35.77)
LDL (mg/dL)	116.086 (31.01)	>130	926 (30.06)
Total cholesterol (mg/dL)	194.57 (34.89)	≥240	343 (10.46)
Triglycerides (mg/dL)	113.45 (84.63)	≥150	592 (19.00)
Waist to hip ratio	0.80 (0.07)	≥0.85	798 (24.53)
Glucose level (mg/dL)	98.08 (31.09)	≥110	341 (10.93)
CRP (mg/L)	3.90 (6.26)	>3	1116 (34.39)
DHAS (ug/dL)	129.77 (78.95)	>240	301 (9.12)
History of medication to control metabolic diseases and hypertension	Yes	Yes	129 (3.92)

* SBP: systolic blood pressure; DBP: diastolic blood pressure; HDL: high density lipid protein; LDL: low density lipid protein; CRP: C-reactive protein; DHAS: dehydroepiandrosterone sulfate.

**Table 2:** Distribution of AL scores and AL category.

AL Score	Number	Percentage
0	888	29.45
1	754	25.01
2	555	18.41
3	375	12.44
4	227	7.53
5	118	3.91
6	66	2.19
7	22	0.73
8	9	0.3
9	1	0.03
AL category	Number	Percentage
1 (AL score = 0)	888	29.45
2 (AL score = 1)	754	25.01
3 (AL score = 2–3)	930	30.85
4 (Al score = 4–9)	443	14.65

**Table 3:** Demographic, healthy behaviors, socioeconomic status, and support by AL category.

	Category 1	Category 2	Category 3	Category 4	*p*-Value
	*n* = 888	*n* = 754	*n* = 930	*n* = 443	
Age, Mean (SD)	45.75 (2.67)	45.68 (2.61)	45.99 (2.70)	46.12 (2.82)	0.004
Race/ethnicity, N (%)					
African American	152 (17.12)	206 (27.32)	323 (34.73)	173 (39.05)	
Chinese American	90 (10.14)	57 (7.56)	56 (6.02)	20 (4.51)	
Japanese American	120 (13.51)	76 (10.08)	55 (5.91)	11 (2.48)	
White	492 (55.41)	358 (47.48)	392 (42.15)	185 (41.76)	
Hispanic	34 (3.83)	57 (7.56)	104 (11.18)	54 (12.19)	<0.001
Ever smoked regularly, N (%)					
No	547 (62.30)	448 (59.57)	510 (55.14)	214 (49.20)	
Yes	331 (37.70)	304 (40.43)	415 (44.86)	221 (50.80)	<0.001
Ever drank alcohol regularly, N (%)					
No	347 (39.08)	334 (44.30)	484 (52.04)	254 (57.34)	
Yes	541 (60.92)	420 (55.70)	446 (47.96)	189 (42.66)	<0.001
Leisure physical activity, N (%)					
No	162 (18.39)	174 (23.23)	309 (33.77)	164 (37.10)	
Yes	719 (81.61)	575 (76.77)	606 (66.23)	278 (62.90)	<0.001
Self-rated sleep quality, N (%)					
Sound and restful	373 (42.19)	304 (40.37)	331 (35.90)	146 (32.96)	
Average	364 (41.18)	312 (41.43)	369 (40.02)	183 (41.31)	
Restless	147 (16.63)	137 (18.19)	222 (24.08)	114 (25.73)	<0.001
Family total income, N (%)					
<20 k per year	71 (8.21)	83 (11.31)	171 (19.02)	110 (25.40)	
20–50 k per year	259 (29.94)	245 (33.38)	325 (36.15)	157 (36.26)	
50–100 k per year	350 (40.46)	288 (39.24)	304 (33.82)	135 (31.18)	
≥100 k per year	185 (21.39)	118 (16.08)	99 (11.01)	31 (7.16)	<0.001
Health insurance, N (%)					
No	51 (5.76)	51 (6.76)	103 (11.08)	40 (9.05)	
Yes	835 (94.24)	703 (93.24)	827 (88.92)	402 (90.95)	<0.001
Cancer status, N (%)					
No	847 (95.38)	715 (94.83)	894 (96.13)	410 (92.55)	
Yes	41 (4.62)	39 (5.17)	36 (3.87)	33 (7.45)	0.037

**Table 4:** Associations between AL category and overall cancer risk.

AL Category	Unadjusted (HR, 95% CI)	Model 1 * (HR, 95% CI)	Model 2 ^#^ (HR, 95% CI)	Model 3 ^@^ (HR, 95% CI)
1	reference	reference	reference	reference
2	1.12 (0.72, 1.73)	1.19 (0.77, 1.85)	1.25 (0.80, 1.96)	1.27 (0.82, 1.99)
3	0.83 (0.53, 1.30)	0.93 (059, 1.47)	0.99 (0.62, 1.59)	0.99 (0.61, 1.58)
4	1.64 (1.04, 2.59)	1.88 (1.17, 3.02)	2.09 (1.29, 3.41)	2.08 (1.26, 3.42)
P for trend	0.224	0.072	0.031	0.040

* Mode 1: adjusted by demographic variables (age and race/ethnicity). ^#^ Model 2: Adjusted by demographic variables (included in Model 1) and healthy behaviors (smoking, alcohol consumption, leisure physical activity, and sleeping quality). ^@^ Model 3: Adjusted by demographic variables (included in Model 1), healthy behaviors (included in model 2), and socioeconomic status (family income and health insurance).

**Table 5:** Association between individual biomarkers of AL scores and overall cancer risk.

	HR * (95% CI)
Higher SBP	1.07 (0.63, 1.82)
Higher DBP	0.89 (0.52, 1.53)
Higher HDL	1.33 (0.96, 1.85)
Higher total cholesterol	1.38 (0.87, 2.20)
Higher triglycerides	1.68 (1.16, 2.43)
Higher waist to hip ratio	1.38 (0.95, 1.99)
Higher glucose level	1.24 (0.74, 2.06)
Higher CRP	1.42 (1.01, 2.01)
Higher DHAS	0.75 (0.40, 1.39)
History of medication to control metabolic diseases and hypertension	1.58 (0.79, 3.16)

* Adjusted by demographic variables, healthy behaviors, and socioeconomic status.

## Data Availability

The data that support the findings of this study are available from the corresponding author upon reasonable request.

## Supplementary Materials

The following supporting information can be downloaded at: https://www.mdpi.com/article/10.3390/cancers14133044/s1, Table S1: List of cancer cases.
