# Supplementary material for: Association of Allostatic Load and All Cancer Risk in the SWAN Cohort
Source: Cancers (Basel). 2022 Jun 21;14(13):3044. doi: 10.3390/cancers14133044 (PMC9264860; doi:10.3390/cancers14133044)
Supplement: Supplementary file 1 [file cancers-14-03044-s001.zip › cancers-1679034-supplementary.pdf]

Table S1. List of cancer cases.

| Site | Number | Percentage | Cumulative Percentage |
|---|---|---|---|
| Bone | 1 | 0.62 | 0.62 |
| Brain | 2 | 1.24 | 1.86 |
| Breast | 77 | 47.83 | 49.69 |
| Cervix | 12 | 7.45 | 57.14 |
| Colon | 7 | 4.35 | 61.49 |
| Kidney | 3 | 1.86 | 63.35 |
| Leukemia | 4 | 2.48 | 65.84 |
| Liver | 1 | 0.62 | 66.46 |
| Lung | 4 | 2.48 | 68.94 |
| Melanoma | 1 | 0.62 | 69.57 |
| Other | 22 | 13.66 | 83.23 |
| Ovary | 7 | 4.35 | 87.58 |
| Rectal | 1 | 0.62 | 88.2 |
| Skin | 2 | 1.24 | 89.44 |
| Throat | 6 | 3.73 | 93.17 |
| Uterus | 10 | 6.21 | 99.38 |
| liver | 1 | 0.62 | 100 |
| Total | 161 | 100 | |
